# Defining success in functional cure for chronic hepatitis B: a nationwide survey of physician benchmarks to guide clinical practice and trial design

**DOI:** 10.3389/fpubh.2026.1707447

**Published:** 2026-03-11

**Authors:** Shan Ren, Yisi Liu, Xiao Lin, Junefeng Lu, Lina Ma, Sujun Zheng, Xinyue Chen

**Affiliations:** First Department of Liver Disease Center, Beijing Youan Hospital, Capital Medical University, Beijing, China

**Keywords:** benchmarks, China, chronic hepatitis B, clinical trials, functional cure, physician preferences, survey, treatment guidelines

## Abstract

**Background:**

While functional cure (FC) is the elusive endpoint of chronic hepatitis B (CHB) therapy, a clear consensus on its practical expectations is lacking. To inform the development of new treatments and clinical guidelines, we conducted a nationwide survey to quantify Chinese physicians' perceptions of FC and their benchmarks for successful novel therapies.

**Methods:**

In this cross-sectional study, we administered a detailed online questionnaire to 151 attending physicians and above with extensive experience treating CHB. A quota sampling method was employed to ensure a geographically balanced cohort representative of practice patterns across China.

**Results:**

A total of 151 physicians were surveyed, with the vast majority (70.8%) endorsing functional cure (FC) as the ultimate treatment goal. The most valued clinical benefit of FC was the reduction in liver cirrhosis and hepatocellular carcinoma (mean score 9.6/10). Combination therapy containing Peg-IFNα was favored by 76.1% of respondents as the preferred strategy to achieve FC. Post-treatment, physicians strongly recommended a minimum of one year of follow-up and adjunct consolidation therapy to mitigate relapse risk. Critically, a consensus emerged on key benchmarks for novel therapies: a minimal acceptable FC rate of 30% and a strong preference for regimens based on Nucleos(t)ide Analogs (NAs) and/or Peg-IFNα.

**Conclusions:**

Our findings translate the collective expertise of Chinese physicians into actionable benchmarks for HBV functional cure. The consensus on a minimal 30% cure rate and a preference for combination therapy provide crucial guidance for clinical trial design and the development of novel antiviral strategies.

## Introduction

The World Health Organization (WHO) has established an ambitious target to eliminate viral hepatitis as a public health threat by 2030 (1, 2). As the country bearing the highest burden of hepatitis B virus (HBV) infection, China faces substantial challenges in achieving this goal. While nationwide vaccination programs have successfully reduced HBV endemicity, prompting WHO to reclassify China from high to moderate endemicity (3, 4), the country's large population means an estimated 70 million people still live with chronic HBV infection (5). Current first-line treatments for chronic hepatitis B (CHB) include nucleos(t)ide Analogs (NAs) and pegylated interferon-alpha (Peg-IFNα), with the latter being less frequently utilized in clinical practice. Since 2010, growing evidence from studies investigating optimized combination strategies—such as sequential, add-on, or prolonged intermittent therapy with NAs and/or Peg-IFNα–has demonstrated improved rates of hepatitis B surface antigen (HBsAg) seroclearance, ranging from 14.4% to 44.7% (6, 7). These advances have established functional cure (FC) as both a feasible and desirable treatment endpoint, characterized by sustained HBsAg loss or seroconversion, undetectable HBV DNA, and normalization of liver function (8–10).

Nevertheless, FC remains achievable for only a minority of the broader HBV-infected population, highlighting a pressing need for novel therapeutic approaches that can deliver higher FC rates. Although numerous investigational agents are undergoing clinical trials with primary endpoints focused on HBsAg seroclearance, neither academic societies nor regulatory authorities have established a formal consensus regarding thresholds for clinically meaningful HBsAg clearance rates. To address this critical gap, we conducted a nationwide survey of experienced Chinese physicians specializing in HBV treatment. This study aims to establish clinically-informed benchmarks—including minimal acceptable FC rates and preferred treatment strategies—based on physician perspectives. Our findings provide empirically-derived guidance for the design of clinical trials and evidence-based recommendations for integrating novel therapies into future treatment paradigms.

## Methods

### Study design and sampling

This study was a non-interventional, cross-sectional physician survey conducted using a pre-validated digital questionnaire. To mitigate sampling bias inherent in voluntary response designs and to enhance national comparability, a multistage stratified quota sampling strategy was employed. Geographic stratification was predefined according to the population distribution reported in the Sixth National Population Census of the People's Republic of China (2010) (11), with recruitment quotas allocated to Eastern (42%), Central (31%), and Western (27%) China. The study protocol was reviewed and approved by the Research Ethics Committee of Beijing You'an Hospital, Capital Medical University [Approval No: (2021)046]. All procedures complied with the Declaration of Helsinki. Digital informed consent was obtained from all participants prior to questionnaire access. Further methodological details are presented in Supplementary Table S1.

### Participants and recruitment

Participant selection was designed to target physicians with substantial experience in chronic hepatitis B (CHB) management. A tiered inclusion strategy was applied: (i) Institutional eligibility: Participants were recruited from 89 Grade A tertiary hospitals across 31 provinces, autonomous regions, and municipalities in mainland China; (ii) Physician eligibility: Eligible respondents were attending physicians or above (predominantly associate chief or chief physicians) who reported managing an active caseload of ≥ 50 CHB patients per month.

Recruitment was conducted between September and October *2021* through the IQ*VIA* Clinical Investigator Panel, a professional physician research network. Electronic invitations were distributed to 212 eligible physicians, followed by a standardized reminder telephone call to *non*-responders after a 7-*day* interval. Ultimately, 151 physicians completed the questionnaire and met all quality-control criteria, yielding a verified participation rate of 71.2% (151/212).

### Questionnaire development and instrument validation

The structured questionnaire was developed through an iterative design process. Initial content was informed by a comprehensive literature review and refined through a pilot validation phase with a focused panel of CHB clinical experts to ensure clarity, relevance, and construct validity. The final instrument comprised four thematic domains: (i) Demographics and Practice Capacity: Geographic location, professional title, years of clinical practice, and CHB patient volume managed per month; (ii) Perceptions of Functional Cure: Views on the clinical importance and urgency of functional cure, perceived long-term benefits of sustained viral suppression, and barriers to off-treatment monitoring; (iii) Current Treatment Preferences: Decision-making regarding add-on or switch strategies (e.g., interferon-α or potent nucleos(t)ide Analogs), consolidation therapy duration, and relapse management; (iv) Benchmarking of Novel Therapies: Expectations for new biologic drug (NBD) development, including minimum acceptable functional cure rates (efficacy floor) and preferred treatment duration defining a “successful” therapy. The full questionnaire (all survey items and response options) is provided in Supplementary Table S2.

### Data integrity and statistical quality control

To ensure the robustness of benchmark estimates within this focused expert cohort, a target sample size of >150 completed questionnaires was prespecified, allowing for an approximate 8% margin of error at a 95% confidence level for proportion estimates. Strict quality checks were enforced on all submitted forms. Questionnaires exhibiting logical drift, incomplete dataset termination, or speed-running (duration < 3 SD below mean time) were permanently excluded. Specifically, 9 invalid entries were purged, retaining a clean effectiveness analysis set (*n* = 151). Statistical analyses were performed using R software (version 4.1.1). Descriptive statistics were summarized as mean ± standard deviation (SD) for continuous variables and as frequencies and percentages for categorical variables. Group comparisons were conducted using the chi-square test or Fisher's exact test, as appropriate. All statistical tests were two-sided, with *p* < 0.05 considered statistically significant.

## Results

### Physician characteristics

The recruitment utilized targeted invitations sent to 212 eligible specialists. Following strict quality control, 151 valid responses were retained, yielding an effective response rate of 71.2%, which underscores the high relevance and engagement of the selected panel. Among the 151 participating physicians, there were 72 males (47.7%) -and 79 females (52.3%), with a mean age of 46.1 ± 8.8 years. The cohort covered a broadly representative geographic distribution across China: 42.4% (64/151) from Eastern, 31.1% (47/151) from Central, and 26.5% (40/151) from Western regions. The majority of participants were advanced clinicians; 72.8% held a specific master's or doctoral degree, and 65.6% held senior titles (Associated Chief Physician or Chief Physician). Professionally, the participants were highly experienced specialists primarily affiliated with the Departments of Infectious Diseases (48.3%) and Hepatology (35.8%). The remaining 24 physicians (15.9%) were from other relevant clinical departments, such as Gastroenterology and Internal Medicine. On average, the physicians reported 19.7 ± 8.7 years of experience in treating Hepatitis B (CHB) and managed a high volume of approxmately 188 CHB patients per month. Notably, 100% of participants confirmed prior experience administering interferon-based therapies (Table 1).

**Table 1:** Baseline characteristics of the 151 physicians participating in the online survey, stratified by geographic region.

**Characteristic**	**Total (*n* = 151)**	**East (*n* = 64)**	**Central (*n* = 47)**	**West (*n* = 40)**
**Sex**, ***n*** **(%)**				
Male	72 (47.7)	33 (48.9)	23 (48.9)	16 (40.0)
Female	79 (52.3)	31 (51.1)	24 (51.1)	24 (60.0)
**Age, years**				
Mean (SD)	46.1 (8.79)	47.2 (9.72)	44.5 (8.31)	46.3 (7.62)
**Age group**, ***n*** **(%)**				
30–40	44 (29.1)	17 (26.6)	17 (36.2)	10 (25.0)
41–50	67 (33.4)	27 (42.2)	21 (44.7)	19 (47.5)
>50	40 (26.5)	20 (31.2)	9 (19.1)	11 (27.5)
**Education level**, ***n*** **(%)**				
Bachelor's degree	41 (27.2)	24 (37.5)	6 (12.8)	11 (27.5)
Master's degree	67 (44.3)	28 (43.8)	23 (48.9)	16 (40.0)
Doctoral degree (PhD)	43 (28.5)	12 (18.7)	18 (32.3)	13 (32.5)
**Experience in treating CHB, years**				
Mean (SD)	19.7 (8.66)	20.9 (9.53)	18.8 (8.25)	18.9 (7.57)
**Years in clinical practice**				
Mean (SD)	20.8 (9.04)	21.9 (9.49)	19.6 (8.68)	20.6 (8.73)
**CHB patients treated per month**				
Mean (SD)	187.8 (129.63)	179.8 (123.92)	183.6 (124.89)	205.8 (144.83)
**Experience of ever using IFN**, ***n*** **(%)**				
Yes	151 (100)	64 (100)	47 (100)	40 (100)
**Department**, ***n*** **(%)**				
Hepatology	54 (35.8)	26 (40.6)	15 (31.9)	13 (32.5)
Infectious Diseases	97 (64.2)	38 (59.4)	32 (68.1)	27 (67.5)
**Professional Title**, ***n*** **(%)**				
Attending Physician	52 (34.4)	20 (31.3)	13 (27.7)	19 (47.5)
Associate Chief Physician	45 (29.8)	20 (31.3)	16 (34.0)	9 (22.5)
Chief Physician	54 (35.8)	24 (37.5)	18 (38.3)	12 (30.0)

SD, standard deviation; CHB, chronic hepatitis B; IFN, interferon.

### Perception on functional cure

**Figure 1:**
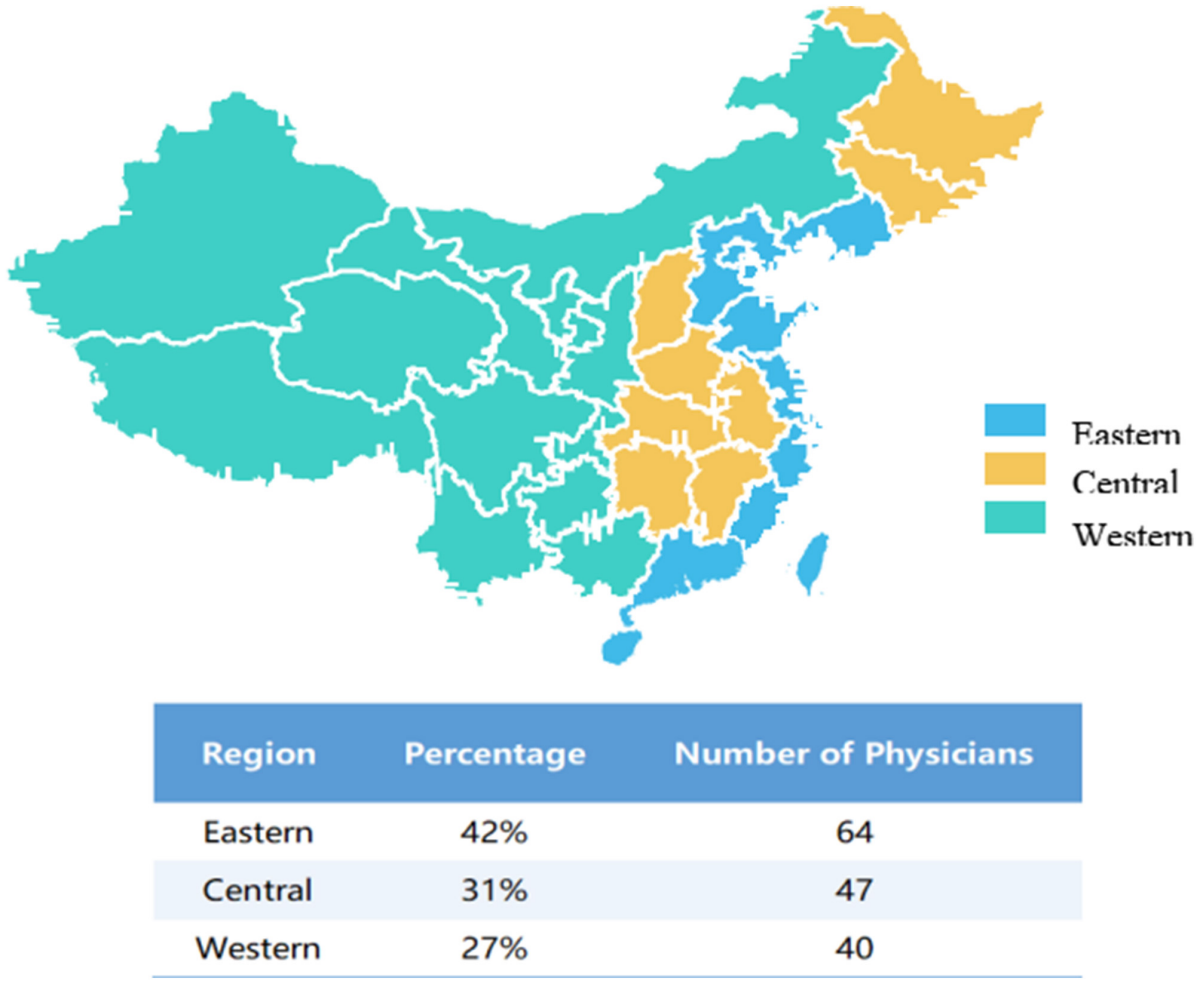
Distribution of physicians across different regions in China.

**Figure 2:**
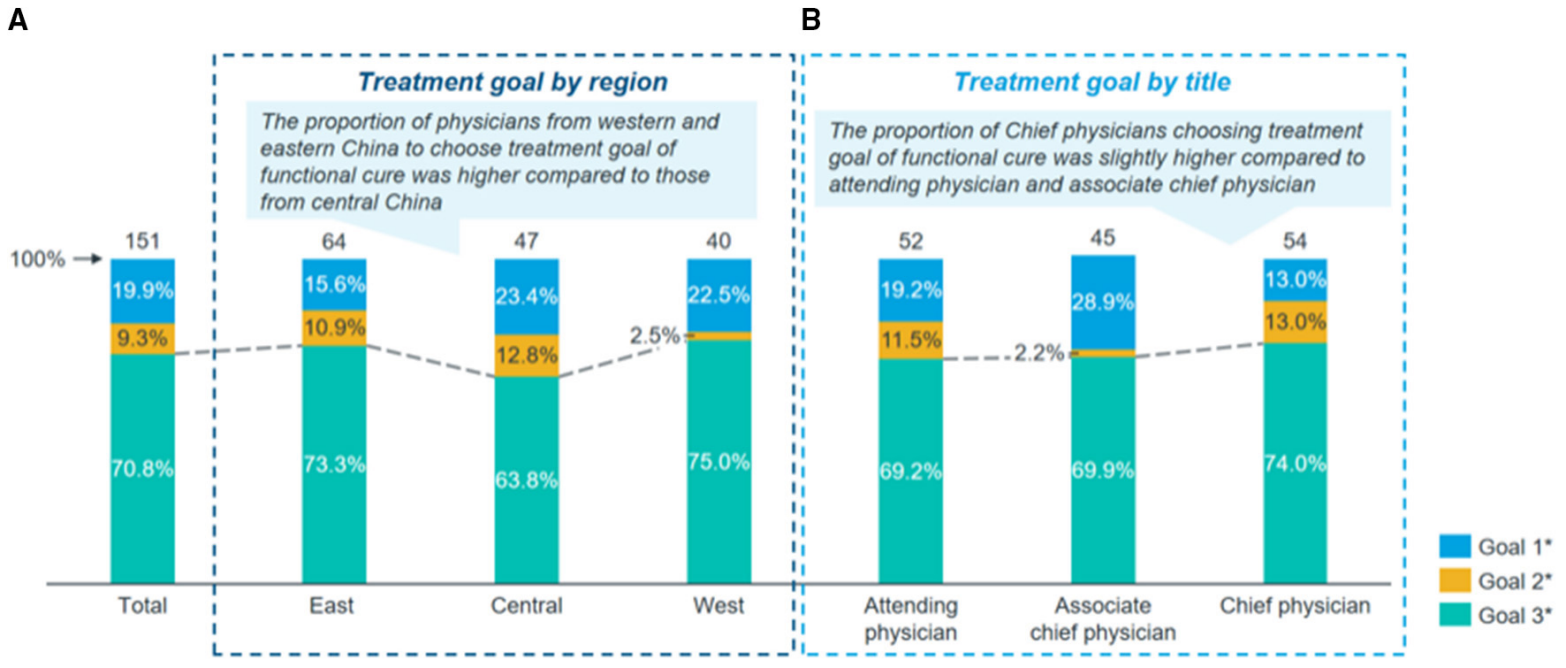
Physicians' perception on functional cure for chronic hepatitis B (CHB): **(A)**. Treatment goal by region: a higher proportion of physicians from Western and Eastern China chose functional cure as the treatment goal compared to those from Central China. **(B)**. Treatment goal by professional title chief physicians showed a slightly higher preference for functional cure as the treatment goal compared to attending physicians and associate chief physicians. Goal 1: Virological and biochemical response – defined as HBV DNA continuously below the detection limit and normalization of ALT^+^. Goal Immune control or inactive carrier status – defined as HBsAg positive (>6 months), HBV DNA < 2000 IU/ml, negative HBeAg or seroconversion of HBeAg, ALT below the upper limit of normal range without ongoing treatment. Goal 3: Functional cure – defined as continuous undetectable HBsAg and HBV DNA after treatment cessation, negative HBeAg, normalization of ALT, and mild or no liver histological lesions. ^+^ALT, Alanine Aminotransferase.

FC was regarded as the ultimate treatment goal by 70.8% of the physicians. Regional differences were observed: 73.3% of physicians from Eastern China, 75.0% from Western China, and 63.8% from Central China endorsed FC as the desired endpoint. By professional title, 74.0% of attending physicians, 69.9% of associate chief physicians, and 69.2% of chief physicians considered FC achievable and ideal (Figure 1). Regarding the clinical benefits associated with FC, reduction in the incidence of liver cirrhosis and hepatocellular carcinoma (HCC) received the highest importance score (mean 9.6), followed by low or no viral relapse after treatment cessation (mean 9.3), based on a 0–10 scale where 10 indicated “very important” (Figure 2). The three most significant barriers to achieving FC were identified as: (1) patient adherence to antiviral therapy, (2) patient socioeconomic status, and (3) limited availability and accessibility of highly effective treatments. Regional variations were noted in perceived barriers; physicians from Eastern China assigned significantly lower scores to “patient intolerance to IFN therapy” compared to those from Central and Western regions (mean scores: 4.5 vs. 5.5 and 6.1, respectively; *p* = 0.002). This gradient, with Eastern physicians perceiving fewer barriers, may reflect greater familiarity and confidence in managing IFN-related adverse events in more developed regions with longer IFN application history. Additionally, chief physicians reported significantly fewer concerns regarding “relative contraindications to IFN” compared to attending and associate chief physicians (mean scores: 4.9 vs. 6.0 and 6.0, *p* = 0.031), reflecting greater confidence in managing IFN therapy (Supplementary Figure 1). The greater confidence among senior physicians suggests that targeted training and knowledge sharing could help mitigate concerns about IFN use among less experienced clinicians.

### Perception of current treatment strategies

**Figure 3:**
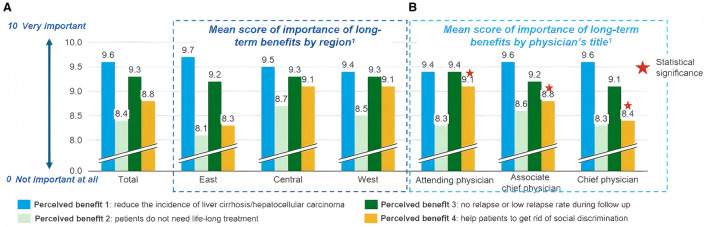
Importance of long-term clinical benefits associated with functional cure, as perceived by physicians. **(A)**. Mean importance scores stratified by geographic region; **(B)**. Mean importance scores stratified by professional title.Benefits were rated on a 10-point Likert scale, where 0 indicates “not important at all” and 10 indicates “very important”:Benefit 1: Reduce the incidence of liver cirrhosis and/or hepatocellular carcinoma; Benefit 2: Patients do not require lifelong treatment; Benefit 3: No or low relapse rate during follow-up; Benefit 4: Help patients overcome social discrimination; Data are presented as mean scores. One-way ANOVA was used to determine, *p*-values for regional and title-based comparisons.

Among the 151 physicians surveyed, 36(23.9%) recommended NA monotherapy for CHB treatment, while the majority(115 physicians, 76.1%) favored combination therapy with NA and Peg-IFNα. Among proponents of combination therapy, 55(47.8%) preferred an add-on strategy, wherein NA was initiated first to achieve sustained virological response before introducing Peg-IFNα. Additionally, 29 physicians (25.2%) supported an individualized approach involving intermittent IFN use alongside NA, guided by HBsAg response and patient tolerance (Figure 3A). Regarding treatment duration, nearly all physicians recommending NA monotherapy endorsed long-term treatment, with a mean duration of 304 weeks. For combination therapy, respondents suggested a mean Peg-IFNα treatment duration of 43–53 weeks, while NA was recommended to be continued for more than 60 weeks as a backbone therapy (Figure 3B). Concerning treatment cessation criteria, FC was considered the ideal endpoint by 62.3% and 72.2% of physicians for HBeAg-positive and HBeAg-negative patients, respectively. Among HBeAg-positive patients, 22.5% adhered to guideline-recommended stopping rules, which require undetectable HBV DNA, normalized ALT, HBeAg seroconversion, and consolidation therapy with NA for 12–36 months. A small proportion of physicians applied similar virological and biochemical criteria with 12–36 weeks of consolidation for HBeAg-negative patients. Furthermore, 15.2% and 18.5% of physicians opposed treatment discontinuation for both HBeAg-positive and HBeAg-negative patients, respectively, to ensure long-term benefits (Supplementary Figure 3). The mean recommended consolidation duration after achieving FC was 43 weeks, with minimal variation across regions or professional titles. Based on physicians' experiential estimates, 68.4% of patients attained virological and biochemical response, whereas only approximately 10.0% achieved functional cure.

### Perceptions on post-functional cure follow-up and relapse in CHB

There was a strong consensus among physicians regarding optimal follow-up strategies after FC, with minimal variation across geographic regions or professional titles. The majority (81%)endorsed a minimum follow-up duration of 24 months, and 58% recommended follow-up intervals of every 3 months after FC achievement (Supplementary Figure 4). Among patients who discontinued treatment after FC, the estimated one-year relapse rate was approximately 5% for HBsAg reappearance alone and an additional 5% for HBV DNA relapse alone. Only 1% of patients experienced both HBsAg and HBV DNA relapse concurrently (Supplementary Table S3). Physicians widely agreed that low anti-HBs titers(90.7%) and insufficient consolidation therapy duration (86.0%) were the two predominant risk factors for viral relapse (Supplementary Figure 5).

As predefined in the study, virological relapse was characterized as HBV DNA > 1,000 IU/ml-with or without HBsAg reversion-on two consecutive tests one month apart. Clinical relapse was defined as virological relapse accompanied by ALT elevation exceeding twice the upper limit of normal (ULN). Among patients who did not achieve FC, virological and clinical relapse rates within one year were nearly 50 and 20%−30%, respectively (Supplementary Table 1). Regarding management strategies for virological relapse (with or without HBsAg reversion), 44.2% of physicians favored reinitiating the original treatment regimen, whereas 30.2% preferred switching to an alternative strategy. Notably, 25.6% recommended watchful monitoring without intervention, particularly in cases with low-level HBV DNA. For clinical relapse, however, the majority (65.1%) advocated switching regimens, while 33.3% supported reusing the original therapy (Supplementary Figure 6).

### Physicians' expectations for novel regimens in CHB treatment

**Figure 4:**
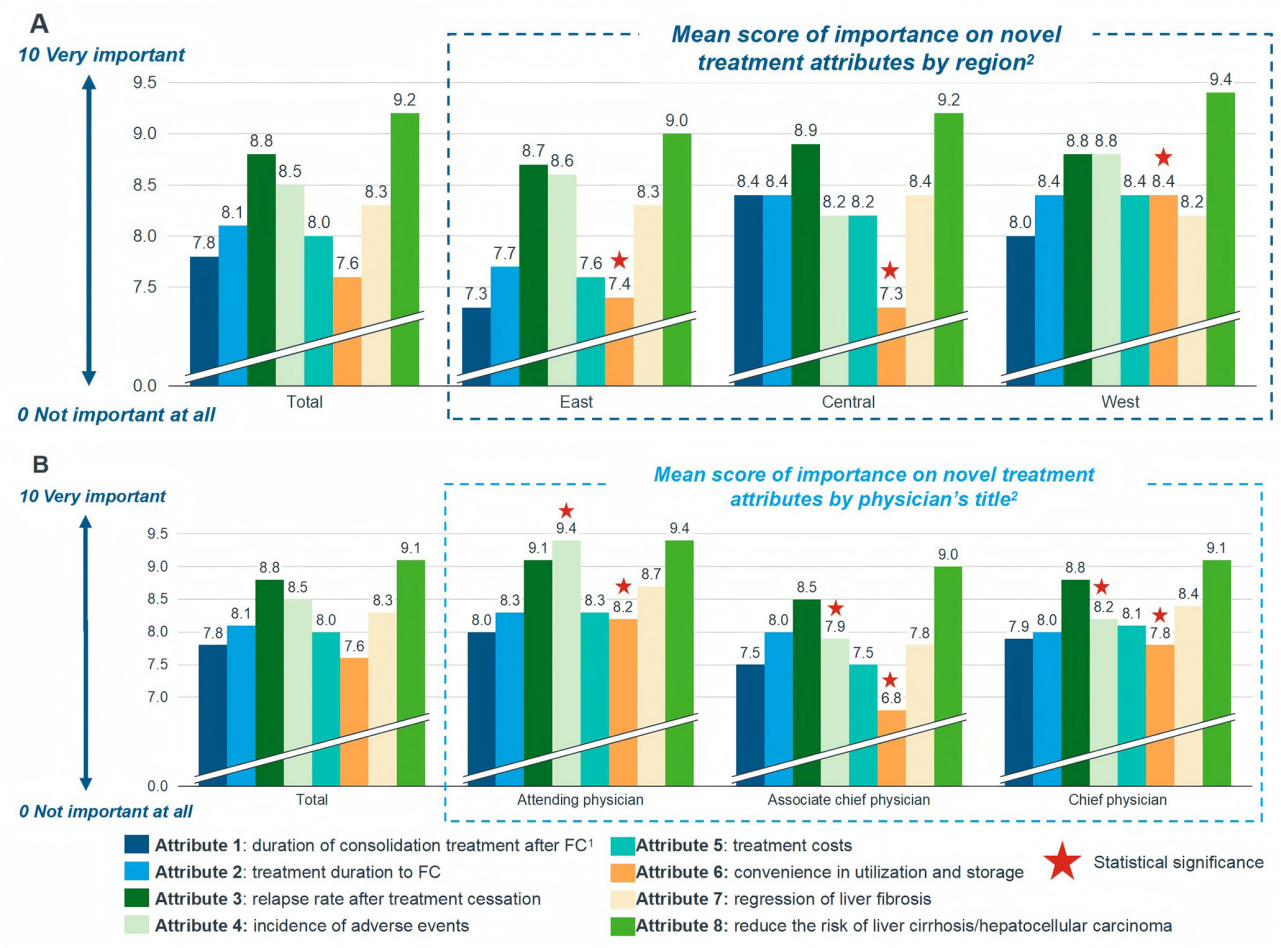
Physicians' perceived importance of attributes for novel chronic hepatitis B (CHB) therapies. Attributes were rated on a 10-point Likert scale (0 = not important at all; 10 = very important). Data are presented as mean scores. **(A)**. Importance ratings stratified by geographic region. Physicians from Western China assigned significantly greater importance to convenience in drug utilization and storage compared to those from Eastern and Central regions (assessed by one-way ANOVA/Kruskal-Wallis test). **(B)**. Importance ratings stratified by professional title. Attending physicians placed significantly higher importance on the incidence of adverse events and convenience of use/storage compared to associate chief and chief physicians (*p* < 0.001 and *p* = 0.001, respectively; one-way ANOVA/Kruskal-Wallis test). FC, functional cure; HCC, hepatocellular carcinoma.

The most valued attributes of novel therapies among physicians were the reduction in the risk of liver cirrhosis and HCC, followed by a lower relapse rate after treatment cessation and minimal adverse effects (Figure 4). In contrast, factors such as the duration of consolidation therapy and convenience of drug use and storage were deemed less critical. Notably, the importance assigned to these attributes varied significantly with physician seniority and geographic region. Attending physicians placed significantly greater emphasis on the incidence of adverse events and convenience of use/storage compared to associate chief and chief physicians (*p* < 0.001 and *p*=0.001, respectively; Figure 4A). This may reflect the practical concerns of less experienced clinicians who are more directly involved in day-to-day patient management and monitoring. Conversely, the importance attributed to novel therapies' capacity for reversing liver fibrosis was positively correlated with longer clinical experience (*r* = 0.200, *p* = 0.013), suggesting that senior clinicians place higher value on histological improvement than their junior counterparts. Geographic variations were also evident. Physicians from Western China assigned significantly greater importance to drug convenience and storage compared to those from Eastern and Central regions (Figure 4B). This regional disparity likely mirrors inequalities in healthcare resources and infrastructure, indicating that implementation strategies for novel therapies may need to be tailored to different socioeconomic contexts across China.

Regarding acceptable thresholds for novel therapies, physicians reported a median minimal FC rate of 30% and a maximum treatment duration of 96 weeks. This physician-defined benchmark of a 30% functional cure rate provides a clinically-relevant and tangible target for phase III trials, suggesting that novel therapies achieving sustained HBsAg loss at or above this threshold would be deemed successful by the treating community. Concerning combination strategies, a large majority (78.1%) favored combination with NAs (maximum duration: 96 weeks), while 39.1% supported combination with Peg-IFNα (maximum duration: 48 weeks), and 38.4% preferred combination with both NAs and Peg-IFNα (maximum duration: 48 weeks), reflecting limited acceptance of Peg-IFNα beyond 48 weeks. This strong preference for NA-based combinations underscores the clinical expectation that novel agents, particularly immunomodulators, should be evaluated on a backbone of potent NA therapy to maximize their therapeutic potential and meet real-world clinical expectations. Only 2.6% of physicians were open to novel agents used without concomitant NA or Peg-IFNα therapy (Table 2).

**Table 2:** Acceptable FC combination therapy and duration for CHB novel treatment.

**Combination therapy**	**Acceptable rate (%)**	**Treatment duration (weeks)**
Combined with NA	78.1	96
Combined with IFN	39.1	48
Combined with NA+IFN	38.4	48
Unacceptable	2.6	–

NA, Nucleos(t)ide Analogs; IFN, interferon.

## Discussion

This nationwide survey yields critical empirical data on the expectations of Chinese hepatologists and infectious disease specialists concerning functional cure (FC) in CHB. The key findings-a consensus that a 30% FC rate is the minimal acceptable threshold for novel therapies, coupled with a pronounced preference for combination strategies incorporating existing agents-establish evidence-based benchmarks that can guide future HBV drug development and treatment policy (12–14).

Our survey also revealed that physicians estimate high virological and clinical relapse rates within one year after treatment cessation in patients who do not achieve FC. These physician-estimated relapse rates are corroborated by long-term follow-up data after Peg-IFN-induced functional cure, which show a concentration of relapse events within the first year (6, 15). They are also aligned with meta-analyses reporting high virological and clinical relapse rates in the first year after treatment cessation in the overall patient population (16, 17). It is critical to note that the relapse risk is highly stratified by the quality of immune reconstitution, particularly the end-of-treatment anti-HBs level, with rates exceeding 50% in patients with anti-HBs < 100 mIU/ml but being below 10% in those with robust antibody responses (6). This highlights the importance of assessing immune status and considering consolidation strategies before treatment cessation.Our results deliver actionable insights for structuring clinical trials and framing real-world therapeutic strategies. For trial design, the consensus around a 30% FC rate provides a clinically grounded target for Phase III trials, aiding in the determination of non-inferiority or superiority margins and facilitating sample size calculation (12, 13, 18). This benchmark aligns with emerging international consensus on feasible and meaningful efficacy thresholds for novel therapies (19, 20). Furthermore, the overwhelming preference for NA-based combination therapy (78.1%) underscores that the developmental paradigm for functional cure is firmly rooted in combination strategies (13, 17). Novel agents, especially immunomodulators, should be evaluated against a backbone of potent NA therapy rather than as monotherapies to meet clinical expectations (13, 17). The accepted treatment duration of up to 96 weeks for these NA-inclusive regimens further delineates a feasible and clinically acceptable time frame for finite-duration trial protocols (6, 8, 10).

For clinical practice, our data inform the development of standardized management protocols for patients who achieve FC with new therapies. The recommended post-FC consolidation therapy (mean 43 weeks) and intensive follow-up strategy (e.g., quarterly in the first year) can directly guide the formulation of clinical guidelines. Moreover, the observed regional variations in perceptions-especially concerning barriers to interferon use and the importance of drug convenience—highlight the necessity for tailored educational initiatives and implementation strategies (14). This will be vital to ensure the equitable and effective adoption of future functional cure therapies across diverse healthcare settings in China (3, 5, 20, 21).

The identification of a 30% minimal acceptable FC rate among experienced Chinese physicians carries significant threefolds implications, extending beyond immediate clinical expectations. For Clinical Trial Design, this physician-derived benchmark offers a pragmatic and clinically meaningful reference for pivotal trials, supplying a robust anchor for powering sample size calculations and establishing non-inferiority or superiority margins (11, 13, 18). A therapy consistently achieving sustained HBsAg loss at or above this rate would represent a transformative advance in the clinical trial landscape. For Health Economic Evaluation, a 30% FC rate constitutes a crucial parameter for cost-effectiveness and budget-impact models. Attaining functional cure in a substantial patient subset could support a premium pricing strategy for curative therapies, justified by the substantial long-term savings from averting lifelong antiviral therapy, monitoring, and management of cirrhosis and hepatocellular carcinoma complications. For Guideline Development, this benchmark aligns with ambitious yet feasible targets proposed by international consortia (18). Integrating this clinician-informed threshold into future iterations of CHB treatment guidelines would ensure that recommendations are not only evidence-based but also reflect realistic clinician expectations, thereby enhancing their acceptability and adoption in real-world practice (8–10).

The strong endorsement of FC by physicians (70.8%) as the ideal treatment goal underscores its central role in contemporary HBV management. This view is consistent with international guidelines (8–10), reflecting a broad global consensus on targeting HBsAg loss. The high priority assigned to FC's potential for reducing the risks of cirrhosis and HCC (mean score 9.6/10) further supports clinical decision-making that emphasizes long-term outcomes over intermediate endpoints. Recent clinical evidence lends robust support to these perspectives. The Piranga study, which reported HBsAg clearance rates aligning closely with the 30% threshold identified in our survey using a novel combination regimen, offers timely validation of these clinical expectations (22). Notably, Professor Hou's innovative strategy combining RNA interference therapy with immune modulation marks a paradigm shift in HBV treatment. In the Piranga study, treatment with xalnesiran (an siRNA therapeutic) plus pegylated interferon-α resulted in an HBsAg clearance rate of 23% at 24 weeks post-treatment, which increased to 47% among patients with baseline HBsAg < 1000 IU/ml (22).

These outcomes are highly consistent with our survey findings, in which 78.1% of physicians indicated a preference for NA-based combination strategies. The promising results from such novel therapeutic platforms confirm that clinician-favored approaches—integrating direct antiviral agents with immune modulators—represent a viable and encouraging pathway toward achieving functional cure. Attributes that may restrict interferon use were further elucidated. For instance, “patient intolerance” was perceived as a more significant barrier among physicians in Central (mean score: 5.5) and Western China (6.1) compared to those in Eastern China (4.5; *p* = 0.004). Similarly, when asked whether “relative contraindications to interferon” posed a barrier, chief physicians reported significantly lower concern (mean score: 4.9) than attending (6.0) and associate chief physicians (6.0; *p* = 0.031). These patterns suggest that physicians in Eastern China—where interferon has been used more extensively—are more familiar with managing its adverse effects, and that senior clinicians exhibit greater confidence in navigating complex clinical scenarios involving interferon. After achieving functional cure, most physicians recommended at least one year of follow-up, with visits every 3–6 months, along with effective consolidation therapy to minimize the risk of virological or biochemical relapse. Furthermore, our survey highlighted that physicians widely recognize younger age (endorsed by 94.1%) and low baseline HBsAg levels (86.1%) as key predictors of treatment success. This convergence between clinical intuition and emerging predictive biomarkers underscores the growing sophistication of HBV management strategies (23, 24).

Our study has several limitations. Its focus on tertiary hospitals may restrict the generalizability of findings to primary care settings, although this aligns with previous specialist surveys (20). The cross-sectional design captures perceptions at a single time point and is susceptible to recall bias, as evidenced in similar international studies (21). Additionally, real-world barriers such as the upfront costs and variable regional availability of Peg-IFNα–determined by local reimbursement policies and hospital formularies—were not evaluated; however, these factors are critical for implementing functional cure strategies despite their established long-term efficacy. In conclusion, this inaugural national survey establishes a physician-defined benchmark of a 30% minimal acceptable functional cure rate alongside a strong preference for combination therapies. These findings offer actionable guidance for clinicians, researchers, and drug developers, thereby paving the way for standardized, evidence-based approaches to achieving functional cures both in China and globally.

## Data Availability

The original contributions presented in the study are included in the article/Supplementary material, further inquiries can be directed to the corresponding author.
