# Supplementary material for: Defining success in functional cure for chronic hepatitis B: a nationwide survey of physician benchmarks to guide clinical practice and trial design
Source: Front Public Health. 2026 Mar 11;14:1707447. doi: 10.3389/fpubh.2026.1707447 (PMC13013281; doi:10.3389/fpubh.2026.1707447)
Supplement: Supplementary file 1 [file Supplementary_file_1.docx]

Table S1. Relapse rate within one year after treatment cessation by physicians’ experience

|  | HBeAg (+) patients | HBeAg (-) patients |
| --- | --- | --- |
| Not achieving FC (%) |  |  |
| Virological relapse | 50 | 50 |
| Clinical relapse | 30 | 20 |
| Achieving FC (%) |  |  |
| HBsAg relapse | 6 | 1 |
| HBsAg, HBV DNA relapse | 5 | 5 |
| HBV DNA relapse | 3 | 1 |

Functional Cure, FC

**
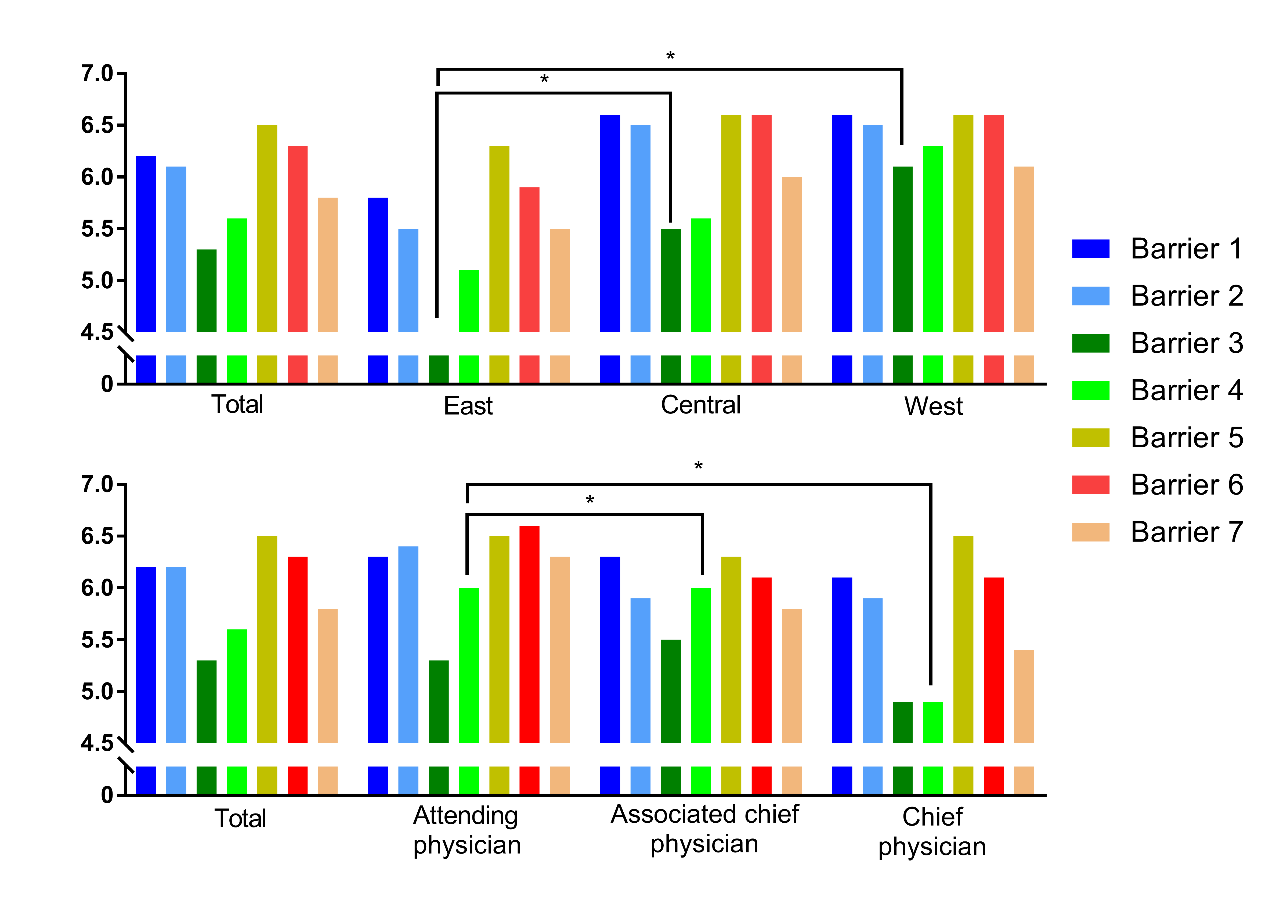
** **Supplementary Figure1.** Mean scores of difficulty on barrier to achieve FC perceived by physicians

Barrier 1: availability of effective treatment. Barrier 2: treatment duration of NA. Barrier 3: patient’s intolerance to IFN treatment. Barrier 4: Contraindication for IFN. Barrier 5: Patient’s treatment compliance. Barrier 6: Patient’s socioeconomic status. Barrier 7: potential to achieve functional cure based on current treatment. One-way ANOVA was used to determine *p* value. * *p*<0.05. (0 means very difficult, 10 means not difficult at all)

**
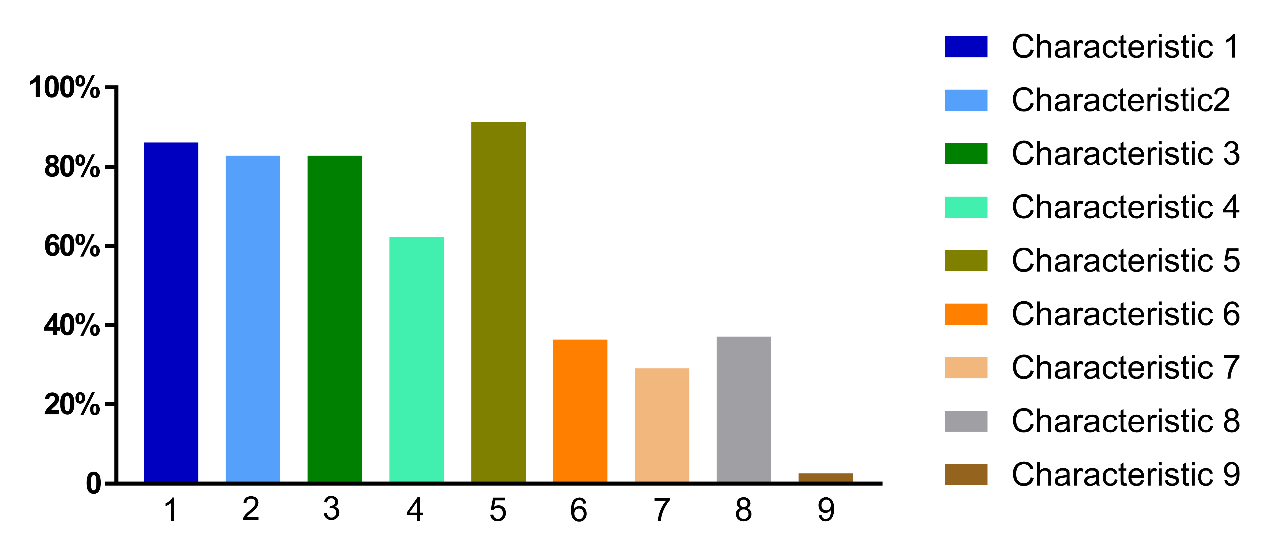
Supplementary Figure 2.** Figure 4. Chracteristics of optimized patients to achieve FC

Characteristic 1: Younger age. Characteristic 2: High baseline ALT. Characteristic 3: Low baseline HBV DNA leve. Characteristic 4: Low baseline HBeAg level. Characteristic 5: Low baseline HBsAg level. Characteristic 6: Treated with NA. Characteristic 7: Treated with IFN. Characteristic 8: HBV genotype B. Characteristic 9: Other.


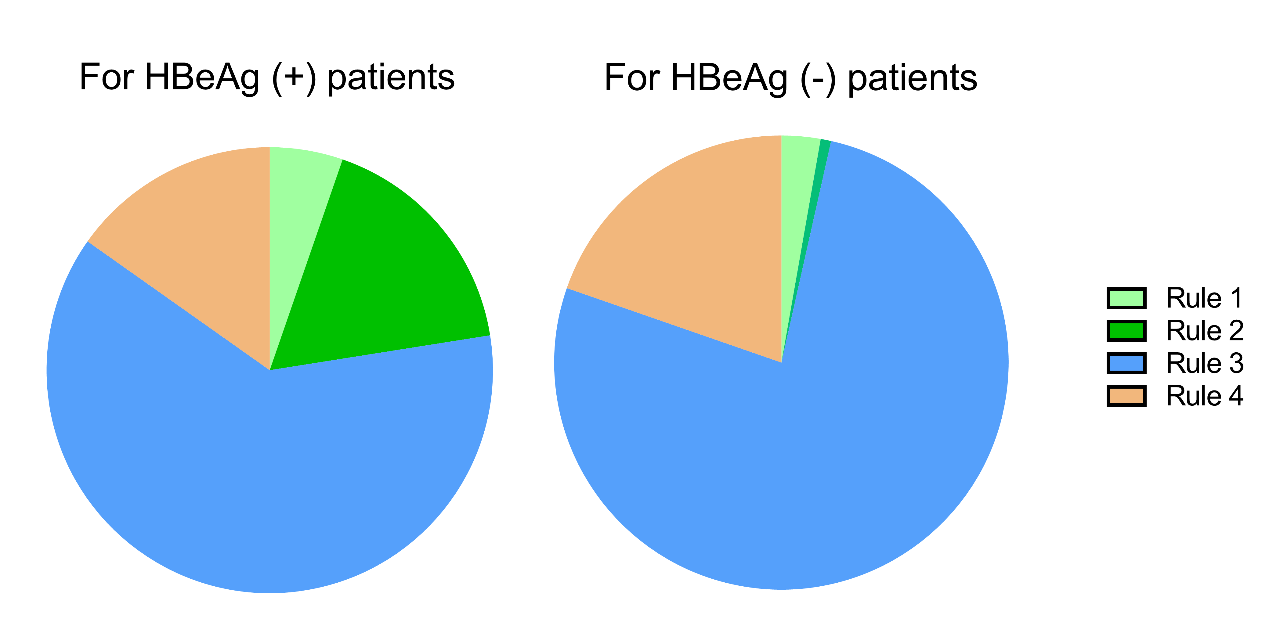
 **Supplementary Figure 3**. Treatment cessation rule for HBeAg (+) and HBeAg(-) patients

Rule 1: Undetectable HBV DNA, normal ALT with at least 12-month consolidation treatment. Rule 2: Undetectable HBV DNA, normal ALT with at least 36-month consolidation treatment. Rule 3: Functional cure. Rule 4: Do not consider treatment discontinuation.


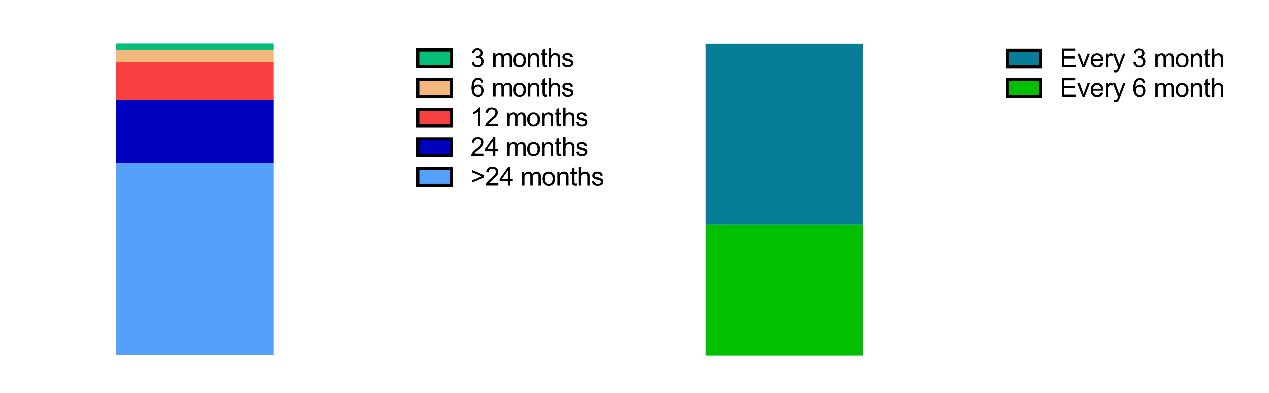
 **Supplementary Figure 4**. Follow-up time and frequency recommended by physician

**
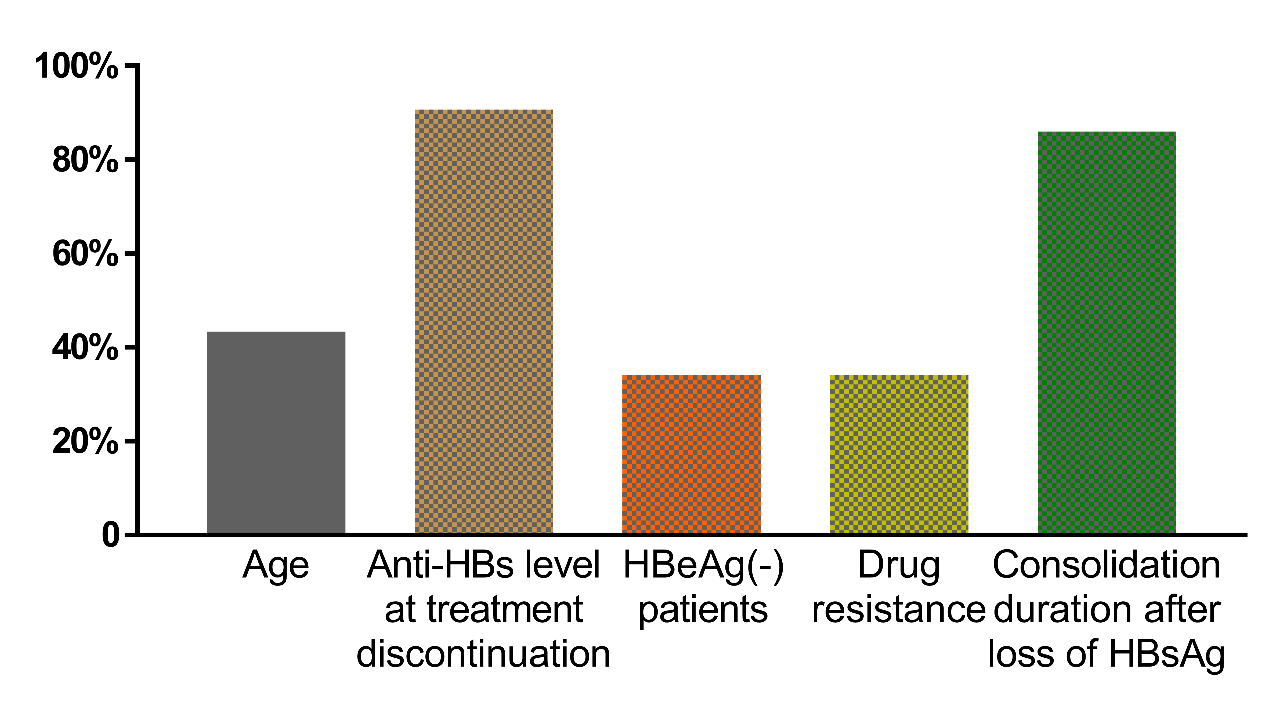
Supplementary Figure 5**. Predictive factors of CHB relapse considered by physicians


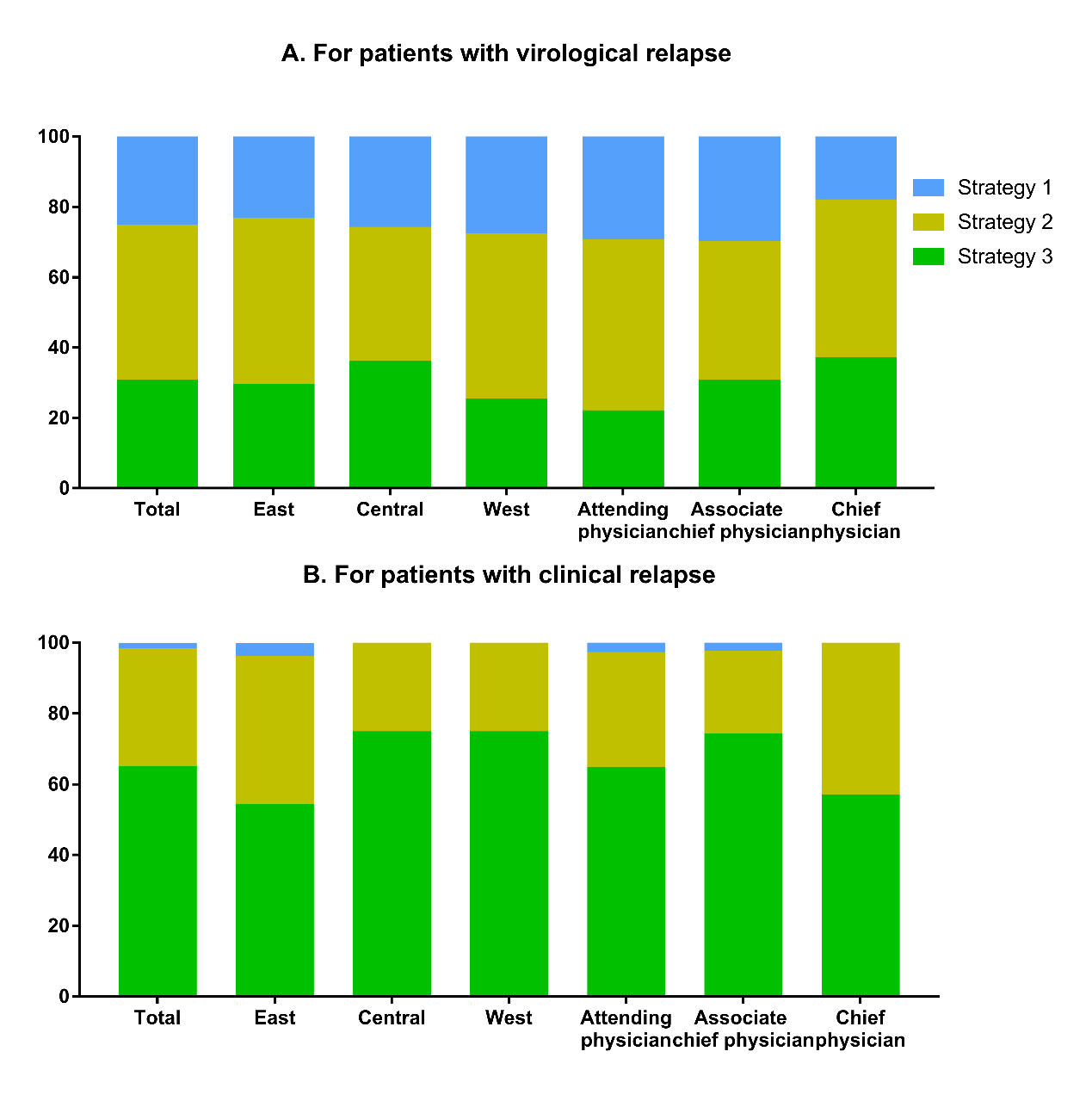
 **Supplementary Figure 6**. Treatment strategy for relapsed patients after treatment cessation

Strategy 1: Continue to follow up. Strategy 2: Switch treatment. Strategy 3: Maintain original treatment.
