# Supplementary material for: Defining success in functional cure for chronic hepatitis B: a nationwide survey of physician benchmarks to guide clinical practice and trial design
Source: Front Public Health. 2026 Mar 11;14:1707447. doi: 10.3389/fpubh.2026.1707447 (PMC13013281; doi:10.3389/fpubh.2026.1707447)
Supplement: Supplementary file 2 [file Table_1.docx]

Physicians were selected via a two-stage sampling approach. Hospitals with strong expertise in infectious disease or hepatology in each of the three regions (East, Central and West) were selected first. Attending physicians or above from the two departments in the selected hospitals were invited to participate in the online survey. If potential participants agree to participate, they can click the web link to answer eligibility screening questions. Eligible physicians were offered an informed consent form (ICF) to sign before answering online survey questions. Reminding phone call were made to physicians who do not respond to the invitation in a week. In each hospital, if three eligible physicians have provided active responses in the first round of survey, no more physicians were reached out in the same hospital. If the final questionnaire has been dropped out due to quality issue, the same amount of questionnaires were re-collected from other eligible physicians.

In terms of statistical consideration, due to operational constrains, this study aims to include responses from 150 physicians into the final analyses. Using the formula below, given 95% confidence level and an assumed 50% acceptance rate, this study can achieve 8% margin of error with 150 physicians. If the actual acceptance rate was either higher or lower than the assumed 50%, this study will achieve a lower margin of error.


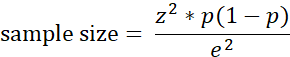


e: margin of error

z: z-score

The sample size under different margin of error as below:

| **Margin of error** | **Sample size** |
| --- | --- |
| **7%** | 196 |
| **8%** | 150 |
| **9%** | 119 |

The potential bias in this study may include: Selection bias. In this study, experienced physicians in grade A tertiary hospitals with expertise in infectious disease or hepatology will be selected. The selection bias may arise that those experienced physicians practicing in hospitals with lower level could be under-represented. But no evidence indicates difference on the perceptions of CHB treatment between experienced physicians from grade A tertiary hospitals and hospitals with lower level. The impact on sample representativeness by this bias could be minimal.

Missing data for each variable were reported (as frequencies and percentages). Where possible, reasons for missing were investigated by summary table with univariate statistics for each variable and logistic regression analysis. To avoid missingness, survey questionnaire was carefully designed with appropriate wording, fit and exhaustive response categories. Questions were set as compulsory and could not be skipped. After the completion of data collection, quality control was conducted on collected questionnaires by well-trained reviewers.
