# Supplementary material for: Defining success in functional cure for chronic hepatitis B: a nationwide survey of physician benchmarks to guide clinical practice and trial design
Source: Front Public Health. 2026 Mar 11;14:1707447. doi: 10.3389/fpubh.2026.1707447 (PMC13013281; doi:10.3389/fpubh.2026.1707447)
Supplement: Supplementary file 3 [file Table_2.docx]

**A physician survey on functional cure for chronic hepatitis B in China**

Q1: What is your gender?

- Male
- Female

Q2: What is your year of birth?

Q3: What is your location?

- East
- Central
- West

Q4: What is your education level?

- Bachelor degree
- Master degree
- PhD and above

Q5: Are you a doctor in a third-class hospital?

- Yes
- No

Q6: What is your department?

- Hepatology
- Communicable disease
- Infectious disease
- Others

Q7: What is your title?

- Resident physician
- Attending physician
- Associate chief physician
- Chief physician

Q8: How many years have you been in clinical practice?

Q9: How many years have you been in treating CHB patients?

Q10: How many hepatitis B patients have you received each month on average in the past six months? (multiple visits by the same patient are counted as 1 case)

Q11: Have you had any experience of using IFN during the treatment for hepatitis B?

- Yes
- No

Q12: What are your ultimate treatment goal for CHB patients?

- Virological and biochemical response (defined as HBV DNA continuously lower than the detection limit and normalization of ALT)
- Immune control or inactive carrier status (defined as HBsAg positive>6 months, HBV DNA<2000IU/ml, HBeAg negative or seroconversion of HBeAg, ALT<the upper limit of normal range without ongoing treatment).
- Functional cure (defined as continuous undetectable HBsAg and HBV DNA after treatment cessation, negative HBeAg, normalization of ALT, mild or no liver histological lesions)

Q13: How important do you think the following clinical benefits of functional cure for CHB patients are? (0 means not important at all, 10 means very important)

- Reduce the incidence of liver cirrhosis/hepatocellular carcinoma
- Patients do not need life-long treatment
- No relapse or low relapse rate during follow up
- Help patients to get rid of social discrimination

Q14: How difficult do you think the following barriers to achieve functional cure of CHB are? (0 means very difficult, 10 means not difficult at all)

- Availability of effective treatment
- Treatment duration of NA
- Patient’s intolerance to IFN treatment
- Relative contraindication for IFN
- Patient’s treatment compliance
- Patient’s socioeconomic status
- Potential to achieve functional cure based on current treatment

Q15: What do you think are the characteristics of optimized patients who can achieve functional cure?

- Younger age
- High baseline ALT
- Low baseline HBV DNA level
- Low baseline HBeAg level
- Low baseline HBsAg level
- Treated with NA
- Treated with IFN
- HBV genotype B
- Other

Q16a: What is your recommended treatment strategy for CHB?

- NA monotherapy
- IFN monotherapy
- Combined therapy (NA in combination with IFN)

Q16b (If Q16a choose combination therapy): What combination therapy do you recommended?

- Concomitant administration of NA and IFN
- Switching from NA to IFN
- Adding IFN to stable NA
- Intermittent use of IFN in combination with NA

Q17: What is the treatment duration for your recommended treatment strategy for CHB based on your clinical experience? (If Q16a choose combination therapy, the treatment duration of NA and IFN need to be written separately)

Q18a: What percentage of patients achieved virological and biochemical response based on your previous experience?

Q18b: What percentage of patients achieved immune control or inactive carrier status based on your previous experience?

Q18c: What percentage of patients achieved functional cure based on your previous experience?

Q19a: What is your treatment cessation rule for HBeAg negative patients?

- Undetectable HBV DNA, normal ALT, HBeAg seroconversion with at least 12-month consolidation treatment
- Undetectable HBV DNA, normal ALT, HBeAg seroconversion with at least 36-month consolidation treatment
- Functional cure
- Do not consider treatment cessation

Q19b: Are there any HBeAg negative patients who meet the treatment cessation rule in your past experiences?

- Yes
- No

Q19c: What is virological relapse (defined as two consecutive HBV DNA>2000IU/ml one month apart) rate within one year after treatment cessation of HBeAg negative patients who not achieved functional cure?

Q19d: What is clinical relapse (defined as virological relapse with ALT level>2 times upper limit of normal) rate within one year after treatment cessation of HBeAg negative patients who not achieved functional cure?

Q19e: What is HBsAg relapse rate within one year after treatment cessation of HBeAg negative patients who achieved functional cure?

Q19f: What is HBV DNA relapse rate within one year after treatment cessation of HBeAg negative patients who achieved functional cure?

Q19g: What is both HBsAg and HBV DNA relapse rate within one year after treatment cessation of HBeAg negative patients who achieved functional cure?

Q20a: What is your treatment cessation rule for HBeAg positive patients?

- Undetectable HBV DNA, normal ALT, HBeAg seroconversion with at least 12-month consolidation treatment
- Undetectable HBV DNA, normal ALT, HBeAg seroconversion with at least 36-month consolidation treatment
- Functional cure
- Do not consider treatment cessation

Q20b: Are there any HBeAg posieive patients who meet the treatment cessation rule in your past experiences?

- Yes
- No

Q20c: What is virological relapse rate within one year after treatment cessation of HBeAg positive patients who not achieved functional cure?

Q20d: What is clinical relapse rate within one year after treatment cessation of HBeAg positive patients who not achieved functional cure?

Q20e: What is HBsAg relapse rate within one year after treatment cessation of HBeAg positive patients who achieved functional cure?

Q20f: What is HBV DNA relapse rate within one year after treatment cessation of HBeAg positive patients who achieved functional cure?

Q20g: What is both HBsAg and HBV DNA relapse rate within one year after treatment cessation of HBeAg positive patients who achieved functional cure?

Q21: How long do you think is recommended duration of consolidation treatment after achieving functional cure? (week)

Q22: How long do you recommended to follow up CHB patients after functional cure?

- 3 months
- 6 months
- 12 months
- 24 months
- >24 months

Q22: What is recommended follow-up frequency for CHB patients within 1 year after functional cure?

- Every 3 month
- Every 6 month
- Every 12 month

Q23: What are the predictors of relapse after treatment cessation based on your experience?

- Age
- Anti-HBs level at treatment discontinuation
- HBeAg negative
- Drug resistance
- Consolidation duration after loss of HBsAg
- Others

Q24a: What are the treatment strategies for patients with virological relapse after treatment cessation?

- Continue to follow up
- Switch treatment
- Maintain original treatment

Q24b: What are the treatment strategies for patients with clinical relapse after treatment cessation?

- Continue to follow up
- Switch treatment
- Maintain original treatment

Q25: What is the least acceptable functional cure rate of CHB novel treatment?

Q26: What is the longest acceptable treatment duration of CHB novel treatment? (week)

Q27: How important do you think the following attributes of CHB novel treatment are? (0 means not important at all, 10 means very important)

- Duration of consolidation treatment after functional cure
- Treatment duration to functional cure
- Relapse rate after treatment cessation
- Incidence of adverse events
- Treatment costs
- Convenience in utilization and storage
- Regression of liver fibrosis
- Reduce the risk of liver cirrhosis/hepatocellular carcinoma
- Others

Q28: What is the acceptable combined therapy if CHB novel drugs need to be combined with an existing drug?

- Combined with NA
- Combined with IFN
- Combined with NA+IFN
- Unacceptable
